# A pilot study of a stepped-care brief intervention to help psychologically-distressed women displaced by conflict in Bogotá, Colombia

**DOI:** 10.1017/gmh.2019.26

**Published:** 2019-12-02

**Authors:** J.M. Shultz, H. Verdeli, Á. Gómez Ceballos, L.J. Hernandez, Z. Espinel, L. Helpman, Y. Neria, R. Araya

**Affiliations:** 1 Center for Disaster & Extreme Event Preparedness (DEEP Center), University of Miami Miller School of Medicine, Miami, FL, USA; 2 Teachers College, Columbia University New York, NY, USA; 3 Universidad de Los Andes, Bogota, Colombia; 4 Universidad de Los Andes, Bogota, Colombia; 5 Department of Psychiatry and Behavioral Health, University of Miami Miller School of Medicine and Jackson Memorial Hospital, Miami, FL, USA; 6 Department of Psychiatry, Columbia University Medical Center, New York, NY, USA; 7 Department of Psychiatry, Columbia University, The New York State Psychiatric Institute, New York, NY, USA; 8 Centre for Global Mental Health, King's College Hospital, Institute of Psychiatry, Psychology, and Neurosciences, London, UK

## Abstract

**Background:**

Colombia's 6.5 million internally displaced persons (IDPs) have been exposed to trauma, loss, and hardships. Common mental disorders (CMDs) are prevalent in this group, yet there are few evidence-based psychosocial interventions for this population. We assessed the feasibility and acceptability of a stepped-care intervention for women IDPs in Bogota, Colombia.

**Methods:**

Feasibility to recruit participants for an intervention trial, to screen for CMDs and displacement-related traumas, to refer high-risk cases to professional consultation, to implement evidence-based interpersonal counseling (IPC) for women with diagnosed CMDs, to retain participants in the intervention, and to conduct follow-up assessments was assessed. Assessment instruments were validated. The intervention was delivered by trained outreach personnel. Intervention acceptability was assessed by monitoring session attendance, dropout rates, and satisfaction. Potential efficacy was evaluated with pre- and post-intervention measures of CMDs.

**Results:**

We recruited 279 women IDPs into the intervention. On screening, 177 (63.4%) had symptom levels suggesting a CMD. Participants endorsed a wide range of displacement-related exposures. Most participants receiving IPC decreased their symptom levels at follow-up. Many participants did not complete the recommended number of IPC sessions; loss to follow-up was 30%. The performance of the outreach personnel improved after the initial intervention team was replaced with community members trained to deliver the intervention. The Bogotá health system was unable to reliably accommodate emergency psychiatric referrals.

**Conclusions:**

The IPC intervention shows promise, but significant challenges remain for improving reach, adherence, and participant retention. We identified strategies and partnerships to redress some of the main study limitations.

## Introduction

After five decades of traumatizing civil war (Reardon, 2018), Colombia has officially identified more than eight million citizens as ‘victims of the armed conflict,’ including 6.5 million internally displaced persons (IDPs) (Chaskel *et al*. 2015*a*, *b*; GRID, 2018). Colombia has consistently ranked either first or second in numbers of conflict-displaced persons for 15 consecutive years (GRID, 2018). Seventy percent of Colombian IDPs are women and children (Shultz *et al*. 2014*a*, *b*). Throughout each phase in the trajectory of forced migration, Colombian IDPs are exposed to extreme adversities (Shultz *et al*. 2014*a*; Ramirez *et al*. 2016) which are frequently linked to high levels of psychological distress (Chaskel *et al*. 2015*a*; Gaviria *et al*. 2016).

Colombia's Law 1448 (Law of the Victims, 2011) identifies IDPs as ‘protected citizens’ eligible to receive medical, mental health, social, and legal services (Chaskel *et al*. 2015*b*). Health services are meant to be delivered by teams of professionals based at primary care clinics and by mobile community health teams that visit remote, underserved neighborhoods. Evidence-based approaches to address trauma and diagnosable psychopathology among IDPs have been generally lacking until recently (Murray *et al*. 2014; Bonilla-Escobar *et al*. 2018).

Studies on mental health needs of Colombian IDPs have shown high prevalence of trauma exposure and common mental disorders (CMDs) such as depression, anxiety, posttraumatic stress disorder (PTSD), and substance use disorders (Shultz *et al*. 2014*a*; Lagos-Gallego *et al*. 2017, 2018; Santaella-Tenorio *et al*. 2018), especially among women (Wirtz *et al*. 2014; Ramirez *et al*. 2016; Restrepo, 2016). In spite of this reality, IDPs have limited access to the already over-burdened Colombian mental health care system and no intervention has yet proven to be efficacious with this population. There are also logistical barriers to accessing services such as lack of transportation or child care options, perception of mental health care as a low priority, mistrust, and stigma (Gomez Ceballos, 2017).

**Fig. 1.**
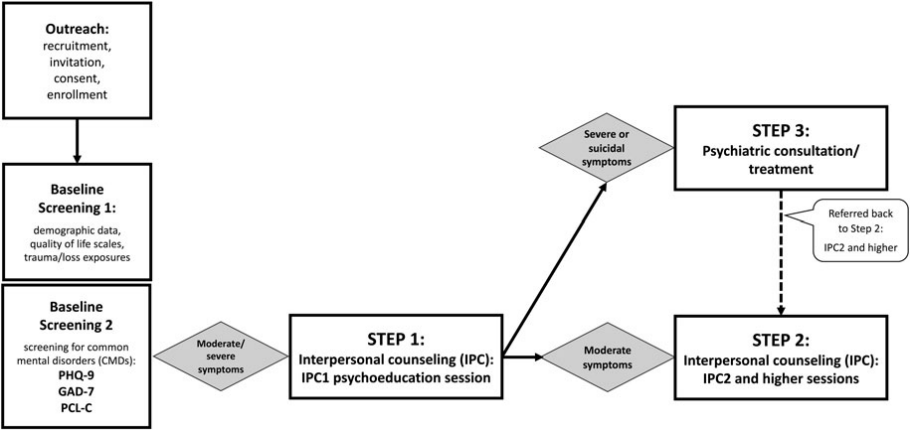
OSITA stepped care model.

To address the dearth of such services, we designed a before-and-after pilot study to examine the feasibility, acceptability, and potential efficacy of an adapted, evidence-based, stepped-care intervention for IDP women with CMDs residing in Bogotá (Fig. 1). The OSITA study, an acronym for Outreach, Screening, and Intervention for TraumA (Shultz *et al*. 2014*c*; Gomez Ceballos *et al*. 2016), was conducted between December 2013 and November 2014 with IDP women residing in Bogotá.

OSITA aimed at examining the feasibility of: (1) *recruiting* sufficient numbers of consenting participants to receive this intervention; (2) *screening* for CMDs [PTSD, major depressive disorder (MDD), and generalized anxiety disorder (GAD)] and displacement-related trauma/loss exposures, using standardized measures; (3) *intervening* using a locally-adapted version of interpersonal counseling (IPC), (4) *retaining* study participants in the intervention until symptom resolution was achieved, (5) *referring* women with moderate or severe symptom levels to specialized services; and (6) *conducting follow-up* assessments of study participants 30 days after completing the intervention assessing symptom changes post-intervention.

## Methods

### Ethics committee approval

Pursuant to the requirements of the funder, Grand Challenges Canada, human subjects’ approval was sought and received from the ethics committee of the Colombian partner, Universidad de Los Andes.

### Setting

This study took place in Bogotá, Colombia in a variety of outreach venues often attended by IDPs including hospitals and government victim service centers.

### Participants

Eligible participants were women residing in Bogotá who self-reported to be displaced ‘victims of the armed conflict.’ Not all participants had completed the lengthy process to receive governmental certification as victims. Exclusion criteria were psychosis, active substance use, or severe medical or cognitive disability.

### Recruitment

Multiple outreach strategies were attempted. First, OSITA counselors accompanied mobile health teams during their visits to households in areas with high concentrations of IDPs to ensure team safety by working with medical personnel known to these communities. Health teams introduced OSITA counselors to eligible participants. Second, public sector hospitals were encouraged to refer IDP women to OSITA. Third, access to IDP women was sought through the network of government victim centers that process, certify, and support IDPs arriving in Bogotá. Fourth, in one Bogotá municipality (Usme), enrollment took place through house-to-house outreach. Fifth, we recruited participants at adult vocational training programs. Sixth, IDP women were approached at a daycare program when they dropped off their children. Seventh, a variety of other community sites were selected for recruitment. Because strategies originally proposed in the application (1–3) generated a few participants, additional outreach approaches (4–7) were introduced to increase enrollment.

### Screening

Consenting participants were screened by OSITA counselors using a modular questionnaire. The questionnaire collected socio-demographic and quality-of-life information and assessed exposures to traumatic events. CMD symptom levels were assessed using three well-known instruments.

The Patient Health Questionnaire (PHQ-9) assesses MDD symptoms (Kroenke *et al*. 2001), consisting of nine questions, each rated on a 0–3 scale according to the severity of the symptoms. Total scores of 0–4 are considered to be subclinical; 5–9 are mild but not clinically significant; 10–14 are of moderate severity; and 15–27 suggest a severe disorder. The PHQ-9 (Cassiani-Miranda *et al*. 2017) and a shortened PHQ-4 version (Kocalevent *et al*. 2014) have been used previously in Colombia with good results.

The Generalized Anxiety Disorder Screener (GAD-7) assesses GAD symptoms (Spitzer *et al*. 2006). The GAD-7 consists of seven questions, each rated on a 0–3 scale, according to the severity of the symptoms. Total scores of 0–4 are considered subclinical, 5–9 are mild but not clinically significant, 10–14 are of moderate severity, and 15–21 suggest a severe disorder.

Finally, the PTSD Checklist-Civilian Version (PCL-C) assesses PTSD symptoms (Weathers & Ford, 1996). The PCL-C is comprised of 17 questions, each rated on a 1–5 scale with total scores of 17–21 considered subclinical, 22–43 mild, 44–49 moderate, and 50–85 severe.

Moderate and severe symptom levels in any of the scales were classified as clinically significant.

During the first 6 months of field implementation, counselors used a pen-and-paper version of the modular questionnaire. During the second 6 months, counselors used a tablet-based version that facilitated data entry. A customized software application developed for OSITA tabulated CMD scores and produced a psycho-education script, along with suggested referral guidance, according to the screening results.

Counselors re-administered the three CMD scales, typically via telephone contact, during a ‘follow-up’ call that was scheduled at least 30 days after the last intervention contact. The follow-up also asked participants about the completion of referrals and current health status.

### Intervention

The psychological intervention selected for OSITA was IPC. IPC is a brief adaptation of Interpersonal Psychotherapy (Weissman & Verdeli, 2012; Markowitz *et al*. 2014; Markowitz *et al*. 2015; Weissman *et al*. 2017). IPC focuses on interpersonal triggers of current distress/depression, mapped onto four categories of life experiences: grief, interpersonal disputes, role transitions, and interpersonal deficits. IPC was introduced based on the finding that most depressed patients who received IPT in primary care attended an average of three sessions (Klerman *et al*. 1987). The efficacy of IPC has been tested in nine randomized controlled trials (Weissman *et al*. 2014).

OSITA employed a three-stage, stepped-care intervention model (Fig. 1).

For participants who scored in the mild or lesser severity range on all three questionnaires at baseline screening, no further action was taken, but they were contacted at a later date for follow-up data assessments.

Participants who scored in the moderate or greater range on any of the screening instruments at baseline immediately received a session of psychoeducation counseling. Psychoeducation is considered to be an introduction to IPC, and therefore, delivery of psychoeducation was regarded as the IPC1 session (STEP 1). Psychoeducation aimed to reduce guilt, give hope that symptoms were treatable, and encourage participants to make their recovery a priority by reducing as many barriers as possible while also mobilizing available personal and other resources for support.

Following psychoeducation, participants with one or more scores in the moderate or severe symptom range were referred and/or encouraged to return for additional IPC sessions (STEP 2).

STEP 3 was designed for IDP women assessed – at baseline or any other session – to have thoughts/intention to self-harm/suicide (based on a positive response to item 9 of the PHQ-9). These women were referred to psychiatric consultation (outside of OSITA) or to Bogotá's psychiatric emergency service (‘Bogotá 123’). During the first months of the intervention, participants scoring in the severe symptom range on either the PHQ-9 or PCL-C were likewise referred to psychiatric consultation (STEP 3).

The original OSITA stepped-care design called for the referral of participants with high severity scores on CMD screening measures, and/or thought or intent to self-harm, to professional or psychiatric consultation. The original option considered was to refer the participants to psychiatrists at a healthcare foundation affiliated with the university partner. This option proved to be unfeasible given the large numbers eligible for referral. The referral pathway was modified and expanded. Ultimately, participants were referred to six different services during the course of OSITA implementation: (1) SISVECOS, the Bogotá health authority's surveillance system for persons with suicide risk; (2) a mental health center on the university's downtown campus; (3) an emotional/trauma care center affiliated with the university's department of psychology and Boston University; (4) a mental health services center at Catholic University; (5) a psychiatrist at a district hospital; and (6) a managed healthcare organization.

However, referrals for psychiatric consultation were quickly discontinued due to the inability of the Bogotá health system to reliably accommodate these referrals (OSITA attempted to obtain referrals using six different services enlisted during the intervention phase). Instead, these individuals were referred to additional IPC sessions while the team tried to secure psychiatric consultation.

At each IPC session, clinical scales that had been in the moderate/severe range at the previous session were re-administered. Participants were referred to additional IPC sessions until the criterion of two consecutive sessions with no moderate/severe symptoms on any CMD measure was achieved.

### OSITA counselors: selection, training, and supervision

OSITA counselors, along with Bogotá government psychosocial professionals working with victim populations, were trained for a week onsite in Bogotá by the US-based IPC master trainer (HV). Subsequently, counselors were supervised weekly, in-person or online, by two IPC-certified professionals, one based in the USA and the other, a Spanish-fluent supervisor, who provided periodic onsite supervision in Bogotá.

Over the course of the study, three types of counselors were hired. The university partner originally stipulated the hiring of four public health *graduate students* to act as OSITA counselors. To increase sustainability, two community-connected *IDP women* were added as counselors. Based on clinical supervisor assessments, both IDP women and three-of-four student counselors achieved competency in IPC delivery (over 70% items on the IPT checklist scored at or above satisfactory level). The counselor who did not reach competency criteria was removed. Following the review of counselor performance 6 months into the implementation phase, OSITA's original contingent of student counselors was replaced with three *experienced outreach workers*, and three medical students (to fulfill university expectations); all of them met competency criteria. The *IDP women* continued for the full duration of the intervention. Counselors personally scheduled STEP 2 IPC sessions at venues and times that were convenient for their participants.

### Retention and follow-up

Counselors kept records of session attendance. The follow-up data collection, conducted primarily via telephone calls, consisted of a short battery of open-ended questions about the completion of professional referrals and current health status, followed by re-administration of the three CMD screening scales (PHQ-9, GAD-7, PCL-C). The original outreach team did not conduct follow-up data collection systematically during the first 6 months of implementation. This oversight was detected and partially corrected. During the final 6 months of the intervention phase and for 3 months thereafter, the new counselor team, supplemented by volunteer staff, conducted follow-up data collection with repeated attempts made to reach study participants. Every effort was made to conduct follow-up sessions using assessors who had no previous contact with the participants being interviewed.

Additional metrics to assess feasibility and acceptability included yield of participant recruitment by site and counselor, completion of psychiatric referrals by high-risk participants, and dropout prior to reaching criterion for IPC completion (two consecutive IPC sessions with no CMD scales in the moderate/severe symptom range). Ongoing clinical supervision was used to track and maintain intervention fidelity.

### Data analyses

Psychometric tests of the screening instruments were conducted including tests of internal consistency (Cronbach's *α*) and construct validity of each instrument through conducting Exploratory Factor Analysis. First, descriptive analyses (means and proportions) were used to examine the baseline measures and changes over time for those ‘treated’ and ‘untreated,’ as there was no control group in this study. Comparisons include baseline against follow-up means for each CMD scale comparing whenever possible ‘treated’ and ‘untreated’ participants. In order to examine symptom changes over time with more depth, we performed a repeated-measure ANOVA on PHQ-9, GAD-7 and PCL-C scores for completers of treatment, defined as those with at least two IPC sessions and follow-up assessment. Per protocol, all these participants had baseline scores in the moderate/severe range on the analyzed scale. This analysis included three timepoints (baseline, last session seen, and follow-up), including 56 participants with depressive symptoms, 47 with generalized anxiety symptoms, and 40 participants with PTSD symptoms.

## Results

### Recruitment

Across 18 recruitment sources, OSITA enrolled 279 women IDPs, who completed the initial assessment from July 2013 to June 2014 (Table 1). Only 48 (17%) participants were recruited using the three original strategies outlined in the research proposal (0 from medical outreach teams, 10 from hospitals, 38 from three victim centers). The most productive approaches entailed direct household outreach in the municipality of Usme (83 participants) and recruitment based at two vocational training programs, Gente Estrategica (79) and SENA (34).

**Table 1.** Participant baseline information and number recruited by site with site description (n  =  279)

Category		*N*	%
Gender	Female	279	100
**Recruitment site**	**Site description**		
Community of USME	Municipality with high density of IDPs	83	29.8
Gente Estrategica	School/training program for indigenous and Afro-Colombian IDPs	79	28.3
Three victim service centers	District registration and service centers for ‘victims of armed conflict’	38	13.6
SENA	Vocational education and training center	34	12.2
Preschool contacts	Pre-kindergarten care for children under 5 years	10	3.6
Two district hospitals	Hospitals affiliated with district health authority	10	3.6
Snowball referral from victims	Referrals from other IDPs	6	2.6
Community of Soacha	Community with high density of IDPs	4	1.4
Three feeding programs	Community kitchens for the indigent	4	1.4
Four other sites		11	3.9
Educational attainment	Less than primary	4	1.43
	Primary completion	76	27.24
	Secondary completion	149	53.41
	Post-secondary technical education	38	13.62
	Professional/higher education	5	1.79
	Unknown/missing	7	2.51
Civil status	Single/separated	140	50.2
	Civil union	87	31.2
	Married	28	10.0
	Widowed	21	7.5
	Unknown/missing	3	1.1
Living accommodation	Daily rental	8	2.9
	Refugee housing	6	2.2
	Room	55	19.7
	Apartment	209	74.9
	Unknown/missing	1	.4
Health insurance	Unknown/missing	22	8.6
	Universal	169	60.6
	Privately paid	86	30.8
Household members	Living alone	14	5.0
	Living with partner and children	132	47.3
	Living with other family members	128	45.9
	Unknown/missing	5	1.8
Ethnicity	Afro Colombian/Indigenous	81	29.0
	Non-minority	198	71.0

OSITA participants were women IDPs with a mean age of 37.4 (13.6) years. About half (53%) had completed secondary education and an additional 18% had vocational or higher education beyond the secondary level. Approximately half of the participants (50%) were single (never married, separated, divorced) and 41% were coupled (civil union: 31%; married: 10%). Twenty-nine percent were indigenous or Afro-Colombian (11% and 18%, respectively).

### Screening

#### Psychometrics of screening instruments

The three instruments had good internal consistency. The internal consistency coefficients (Cronbach's *α*) for the three CMD measures were: 0.81 for nine items for the PHQ-9; 0.82 for seven items on the GAD-7; and 0.91 for 17 items for the PCL-C (*N*  =  279 for each measure).

PHQ-9 items loaded into one common factor that explained 40.4% of the variance. GAD-7 items also loaded in one factor that explained 48.4% of the variance. The PCL-C showed three main factors that together explained 55.20% of the variance. The method used for factor extraction for the PCL-C was principal components analysis. We began by employing an unrotated factor solution of the 17 items, which revealed three factors. Factor 1 explained 41.03% of the variance, factor 2 – 7.61% of the variance, and factor 3 – 6.56% of the variance. The correlation of three factors with each other ranged from 0.46 to 0.73. The method used for factor structure identification was exploratory factor analysis. We first explored orthogonal solutions for the PCL-C and found that the factor solutions were not optimal. Next, we employed an oblique rotation (promax) to the factors to obtain the best structure, given that psychosocial constructs within the PCL-C are correlated.

#### Baseline CMD symptom scores

Half of the participants [142 (50.9%)] had moderate or severe symptom levels for depression at baseline. Four in 10 had moderate or severe levels of GAD symptoms [113 (40.5%)], and posttraumatic stress symptoms [110 (39.4%)]. The corresponding baseline mean scores (s.d.) were: 9.90 (5.92) for PHQ-9, 8.74 (5.24) for GAD-7, and 40.95 (14.33) for PCL-C. Altogether, 177 participants (63.4%) had symptoms in the moderate/severe range for at least one CMD. Among these 177 participants, moderate/severe scores were found on a single CMD measure for 61 participants, on two CMD measures for 44, and on all three CMD measures for 72.

#### Trauma/loss exposures

**Fig. 2.**
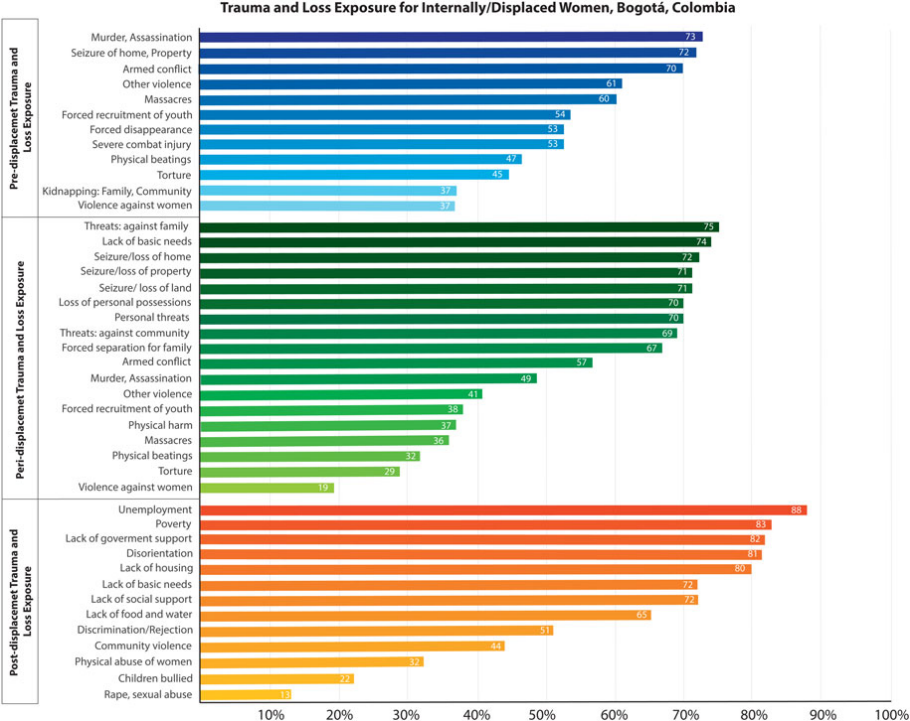
Proportion of IDP women participants (*N*  =  279) endorsing trauma and loss exposures by phase of displacement: pre-displacement, peri-displacement, post-displacement.

At baseline, IDP women (*n*  =  279) endorsed an average of 24.2 of 43 trauma/loss exposures across all phases of forced migration (Fig. 2). This included 6.6 of 12 pre-displacement exposures, 9.8 of 18 peri-displacement exposures, and 7.8 of 13 post-displacement exposures. Pre-displacement exposures featured potentially-traumatizing events such as armed conflict, massacres, kidnapping, and forced-recruitment of children. Peri-displacement exposures around the moment of departure were heavily weighted toward losses (leaving home, possessions, lands, crops and animals, livelihood). Post-displacement exposures included the consequences of relocation to Bogotá (poverty, unemployment, homelessness, lack of urban job skills).

### Intervention

**Fig. 3.**
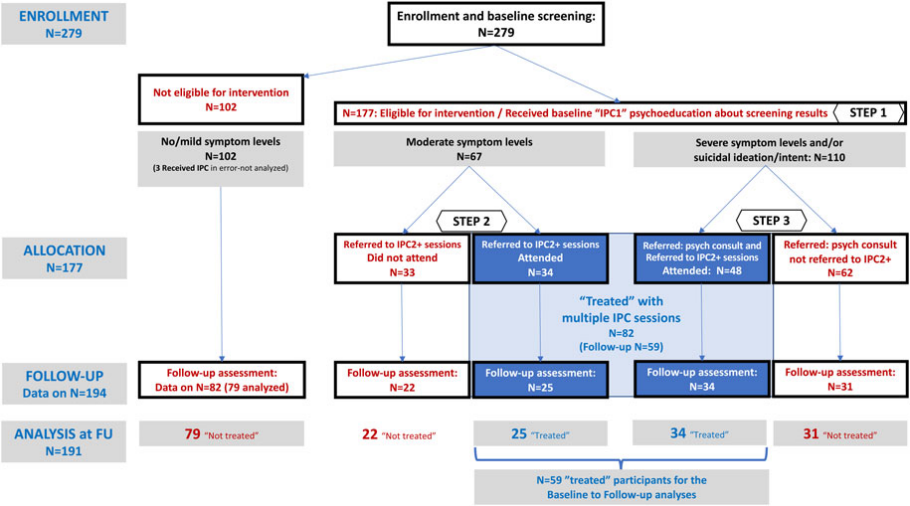
OSITA flow diagram of participants.

As mentioned above, 63.4% of women screened (177/279) were enrolled, the remaining 102 (36.6%) had no/mild symptom levels on all three CMD scales and did not qualify for further action (Fig. 3). Three of these 102 received additional IPC sessions in error; they were removed from the analyses.

All 177 received the IPC1 psychoeducation session (STEP 1) and were referred to either STEP 2 or STEP 3 depending on whether or not they had severe symptoms and/or expressed suicidal ideas (see Fig. 1). Among these, 67 had moderate symptoms but no suicidal thought/intent and were referred directly to STEP 2. The other 110 intervention-eligible participants were initially referred to STEP 3 for psychiatric consultation. While 44 of the 110 had severe symptom levels but no suicidal ideation, the other 66 were referred to STEP 3 because of suicidal ideation (and 54 of these 66 also had severe symptom levels). All women with suicide risk were referred for consultation to one or more of the six referral pathways; however, only 26.1% indicated that they successfully completed the referral.

A total of 82 participants attended the STEP 2 IPC2 session (most continued to IPC3 and higher sessions). The OSITA flow diagram (Fig. 3) shows that 34 of the 67 (50.7%) participants with moderate symptoms/no suicidal ideation who were referred directly to STEP 2 attended the IPC2 session. The other 48 who attended the IPC2 session were participants originally referred to STEP 3 who were referred back to STEP 2 while waiting for psychiatric consultation; this group represented 43.6% of 110 referred to STEP 3. All women with possible suicide risk were referred for consultation to one or more of the six referral pathways; however, only one in four (26.1%) indicated that they successfully completed the referral.

#### Follow-up data collection and analyses

Follow-up measures were completed at least 30 days after the final IPC session; some participants were followed up months later due to the delayed start of follow-up data collection (Fig. 3). Follow-up data collection was obtained via phone call and included questions regarding completion of referrals and self-reported present health status. A total of 204 full or partial interviews were completed (73.1% of 279 participants). Among these, 194 (69.5% of 279) follow-up interviews included a re-administration of the three CMD scales. Follow-up assessments were completed for 82 of 102 (80.4%) participants who had mild or lesser symptom levels on all CMD measures at baseline; three who erroneously received additional IPC sessions were removed from analyses (Fig. 3). The remaining 112 of 194 participants with follow-up assessments represented 63.3% of the 177 who had moderate/severe symptoms at baseline. Of these 112, 59 were treated with multiple IPC sessions, while 53 did not attend IPC2 or higher sessions.

#### Baseline symptom severity for those followed and those lost to follow-up

Compared to the 194 participants with complete follow-up measures, the 85 participants who were not followed had higher baseline symptom scores for all three CMDs. For depression, using the PHQ-9, moderate or severe (‘clinically significant’) symptoms at baseline were observed for 50 (58.8%) of the 85 participants without follow-up assessment compared to 92 (47.4%) of the 194 with follow-up data. Corresponding comparisons for generalized anxiety, using the GAD-7, were 48.2% *v.* 37.1%; and for PTSD, using the PCL-C, 52.9% *v.* 33.5%.

#### Baseline to follow-up changes in symptom levels

Data analyses were performed on 191 of 194 participants with follow-up data (three participants were removed because they received IPC sessions in error). These 191 participants included 59 of the 82 (72.0%) who received IPC2 and higher sessions (‘treated’) and 132 who had no further contact with OSITA after STEP 1. Among these 132 with no further contact with OSITA after baseline, 79 (59.8%) were not eligible for further IPC sessions because symptom scores did not reach the moderate/severe range and therefore cannot be considered dropouts. The remaining 53/132 (40.2%) can be regarded as dropouts, including 22 who were eligible for STEP 2 but did not attend, and 31 who were referred to STEP 3 psychiatric consultation but were not seen again except for follow-up.

Among 59 ‘treated’ participants (eligible and received IPC2 or higher sessions) who completed follow-up assessments, scores for all CMD scales dropped significantly between baseline and follow-up. For the PHQ-9 measure, 78.0% scored in the moderate/severe symptom range at baseline; this declined to 13.6% at follow-up (*p* < 0.001) whilst PHQ-9 means (s.d.) decreased from 12.76 (4.25) to 4.56 (4.32) at follow-up (*p* < 0.001).

For the GAD-7 measure, 62.7% scored in the moderate/severe symptom range at baseline; this declined to 15.3% at follow-up (*p* < 0.001). The GAD-7 mean (s.d.) decreased from 10.75 (4.33) to 4.93 (4.33) at follow-up (*p* < 0.001).

For the PCL-C measure, 54.2% scored in the moderate/severe symptom range at baseline; this declined to 17.0% at follow-up (*p* < 0.001). The PCL-C mean (s.d.) decreased from 46.19 (12.10) to 31.86 (12.24) at follow-up (*p* < 0.001).

Analyses of symptom changes in the sample of completers with baseline, last session, and follow-up data points revealed significant decreases over time in CMD symptom levels (Table 2). For 56 participants, PHQ-9 depression symptoms declined significantly from baseline to last IPC session, and declined significantly again from the last session to final follow-up [*F*_(2,54)_  =  92.05, *p* < 0.001, *η*^2^ = 0.77]. For 47 participants, GAD-7 anxiety symptoms declined significantly from baseline to last IPC session [*F*_(2,45)_, *p*  =  0.01, *η*^2^ = 0.63]. For 40 participants, PCL-C PTSD symptoms declined significantly from baseline to last IPC session [*F*_(2,38)_  =  32.27, *p* < 0.001, *η*^2^ = 0.630]. For the GAD-7 and PCL-C, decreased symptom levels persisted from last session through follow-up, but no additional significant decreases were seen between the last session and follow-up assessment.

**Table 2.** Mean differences in symptom scores for three common mental disorder (CMD) scales (PHQ-9, GAD-7, and PCL-C), comparing baseline to last session^1^, baseline to follow-up, and last session^1^ to follow-up for participants assessed at all three time points

CMD Scale	*N*	Measurement interval: assessment time points	Mean difference	s.e.	*p*
PHQ-9 Major depressive disorder	56	From baseline to last session	5.64	0.68	<0.001
		From baseline to follow-up	8.34	0.61	<0.001
		From last session to follow-up	2.70	0.57	<0.001
GAD-7 Generalized anxiety disorder	47	From baseline to last session	5.36	0.68	<0.001
		From baseline to follow-up	6.34	0.75	<0.001
		From last session to follow-up	0.98	0.55	0.08
PCL-C Posttraumatic stress disorder	40	From baseline to last session	14.33	1.88	<0.001
		From baseline to follow-up	16.25	2.43	<0.001
		From last session to follow-up	1.93	2.06	0.36

The best estimate of effect size is based on those individuals who accessed IPC2 or higher (Table 2). There was a significant decrease in symptom score on all scales. As we said, the differences have to be treated with caution as the study was not powered to test any effect size and there were important changes in the procedures for recruitment and follow-up as can be expected in a feasibility study under challenging conditions such as this one.

### Data access

In concert with the Open Science Movement, detailed study databases have been shared with the funder and we support access to our data.

## Discussion

We tested the feasibility and acceptability of a stepped-care intervention developed for a high-risk population of conflict-displaced women (OSITA) in Bogota, Colombia. We found that this intervention could be feasible and was acceptable to most participants provided important adaptations are made to our original model. IDP women participants had high levels of trauma exposure and psychological symptoms, with almost two-thirds presenting moderate or severe symptoms. Overall, the trend was for symptoms to decrease over time among those receiving the intervention. This is important given that a large proportion of participants were referred to specialized mental health services but were unable to access these services in Bogotá.

The IPT intervention has proven effective for mental health conditions that are elevated in humanitarian settings (namely depression comorbid with anxiety and PTSD) in adult women and men, and adolescents who underwent displacement or severe adversities (Bolton *et al*. 2003, 2007; Verdeli *et al*. 2003, 2008). In the present study, both therapists and participants frequently noted that IPC's focus on loss, conflict, and loneliness made it a good fit and relevant to the experiences of the IDP women participants. The main reported barriers, which are also frequently encountered by other studies in similar settings, concern the financial and logistical difficulties of poor, depressed, and highly vulnerable women to access the sessions on a weekly basis while struggling for their own and their family's survival. Use of mental health outreach to the communities, rather than relying on participants to reach the sites might have increased service utilization considerably.

Regarding the prospect for scaling the OSITA intervention, IPC is amenable to training and dissemination. IPC has been successfully trained to paraprofessionals within a structure that provides appropriate and careful supervision by IPT/IPC certified professionals. We have explored partnerships with teaching hospitals, academic centers, and NGOs in Colombia to train a renewable cadre of IPT-certified professionals.

In terms of feasibility, we encountered many challenges. First, recruitment was difficult. The three initial outreach strategies to recruit participants involved established district government programs. Embedding OSITA personnel into the mobile health teams already operating in the field proved to be logistically unworkable. Asking district hospitals to refer participants to OSITA produced a very low yield of recruitment.

Recruiting through governmental centers established to serve victim populations was seriously delayed by political and organizational complexities. Our Bogotá team worked diligently to demonstrate the complementary elements of OSITA that could potentially enhance government psychosocial programs, but stakeholders were minimally receptive. Late in the project, government gatekeepers finally granted time-limited permission to enroll several dozen IDP women, but with restrictions that made delivery of multiple IPC sessions very difficult.

Access to this population proved to be more far complex than originally envisioned by our local collaborators. Added to the natural reluctance of IDPs to trust other people, we encountered multiple layers of local governance barriers and a lack of clear protocols to access and help IDPs.

The most successful modification to increase recruitment was the shift in personnel to focus on community-experienced outreach personnel who could operate safely in higher-yield community venues. This aligns with the only similar published study (Bonilla-Escobar *et al*. 2018). Future studies would benefit from approaching communities directly, circumventing as much as possible the politically complicated system currently in place to assist these populations in high need.

Second, the high prevalence of severe CMD symptoms and suicidal risk increased pressure for referrals to psychiatric consultation services. We were unable to unlock the complex health system in Bogotá that could have handled such referrals. Despite diligent advocacy to identify referral mechanisms (we attempted six separate pathways), only one-quarter of OSITA's high suicide risk participants reported that they received a professional consultation.

In the other similar study reviewed, investigators set up their own specialized clinic to treat severe cases (Bonilla-Escobar *et al*. 2018). Our original stepped care approach would need to be revised. We would need to seek – or create – our own referral resources rather than rely on existing services. One option would be training human resources – community health workers – to handle the milder cases, thus limiting referrals to specialized services to only the highest-risk cases, as done by Bonilla *et al*. (2018). Similar strategies have been effective in other Latin American countries (Araya *et al*. 2003; Fritsch *et al*. 2007) and other parts of the developing world (Chibanda *et al*. 2016; Patel *et al*. 2017).

Screening proved to be feasible and advisable for future research projects with related study populations. Screening worked effectively to document high levels of trauma/loss exposures that these women had experienced throughout all phases of forced migration. Using internationally-recognized CMD screening measures, it was possible to document very high baseline rates of psychopathology. Likewise, these measures were useful for making clinical decisions within a stepped-care model regarding the continuation of IPC sessions. Nevertheless, although OSITA investigators shared the favorable findings regarding the utility of screening, local government stakeholders were not keen to adopt standardized screening for their psychosocial programs for IDP victims. Regulatory and political hurdles would need to be overcome in order to introduce screening among these populations.

The IPC intervention was well received. It is potentially applicable to Colombia's larger, encompassing a population of forced migrants. OSITA's IPC-certified professional trainers observed that their trained and supervised counselors delivered IPC sessions effectively and mostly according to protocols. The IPC intervention consistently decreased CMD symptom levels, beginning with the IPC1 psychoeducation session. Decreases in symptom levels were maintained throughout the intervention and at follow-up, often several months after the last intervention session. A high proportion of IPC recipients with moderate/severe (‘clinically significant’) symptoms at baseline showed full recovery. A similar study using a different therapeutic approach in a different part of Colombia with indigent and Afro-Colombian participants also found promising, though inconsistent, results depending on locations and populations (Bonilla-Escobar *et al*. 2018).

### Limitations

These encouraging findings are offset by several important limitations. This pilot study did not include a control group. It was not designed to test the efficacy of the intervention. A fully-powered randomized controlled trial in the future will address this aim. The difference in data collection methodology for the CMD scales at baseline (in-person interview) and follow-up (telephone contact) could have contributed to the observed marked decreases in CMD scores. OSITA used counselors to conduct baseline assessments and did not have access or funding for independent evaluators. Follow-up data collection was initially delayed, and this led to sizable losses at follow-up, particularly among participants with severe baseline symptom levels. Although we explored the psychometric properties of the instruments used, we did not find proper local validation studies of these measures for use with IDPs in Colombia.

Also, although we examined exposures to trauma, loss, and life change throughout the phases of displacement as well as their impact on the mental health of the participants, we could have dedicated greater focus to the evaluation of the contextual social factors that could negatively affect their mental health in the present, including ongoing sources of community violence and social exclusion (Chaskel *et al*. 2015*a*, *b*).

Participant attrition was a major problem and led to a qualitative study to explore the reasons for participant dropout (Gomez Ceballos, 2017). The study identified obstacles to attendance at multiple IPC sessions: lack of time, lack of bus fare, lack of child care, family interference, failure to receive employer permission, and pervasive stigma and discrimination for seeking mental health support. Some of the OSITA dropout may have occurred due to participants feeling better after several IPC sessions and not returning. Nevertheless, the other comparable study (Bonilla-Escobar *et al*. 2018) also reported high rates of participant drop-out, suggesting the need to further understand and tackle engagement and access barriers to mental health care within this population.

## Concluding comments

As a feasibility and acceptability study, OSITA was useful for identifying promising program components that work well with Colombia's large population of IDP victims. The screening procedure was successful for channeling participants with moderate/severe CMD symptom levels into the IPC intervention, and objectively determining when symptom improvement had been achieved. The IPC intervention was notable for rapidly and consistently reducing CMD symptom levels for those who attended the sessions.

Nevertheless, other program components require reformulation. The main barrier detected was the inability to achieve efficient enrollment of a representative sample of women IDPs. OSITA outreach activities were, by and large, unsuccessful in accessing this population. For those who were recruited, the follow-on problem was the low rate of retention in treatment; this requires rethinking and restructuring the approach to delivering the intervention. The other primary obstacle is systemic and ongoing. Colombia lacks a dependable referral system for those IDPs who are suicidal or severely symptomatic.

Finally, there is a powerful driver for forging ahead: the mental health needs in this population are compelling. Most participants experience prolonged, clinically significant, and debilitating symptoms of CMDs. Learning from our pilot work, we are confident that reaching out to the community directly, rather than going through intermediaries, is worth exploring. An optimistic and serendipitous finding is that IDP women from the community can be trained to effectively assume the counselor role; their firsthand knowledge of forced migration creates rapport and trust among participants.

In Colombia, there are not many strategies for interventions within vulnerable populations. Given the immense number of displaced persons throughout the country, it is necessary to develop and validate multiple interventions. OSITA has the potential to become one of the options for treatment of mental health problems due to two main reasons: (1) the intervention takes little time and is simple and (2) the intervention has the potential to be economical with the right contingent of intervention staffing.
